# Workplace Violence against Residents in Emergency Department and Reasons for not Reporting Them; a Cross Sectional Study 

**Published:** 2018-01-16

**Authors:** Gilava Hedayati Emam, Hossein Alimohammadi, Akram Zolfaghari Sadrabad, Hamidreza Hatamabadi

**Affiliations:** 1 Emergency Department, Imam Hossein Hospital, Shahid Beheshti University of Medical Sciences, Tehran, Iran.; 2 Emergency Department, Imam Reza Hospital, School of Medicine, Kermanshah University of Medical Sciences, Kermanshah, Iran.; 3 Professor of Emergency Medicine, Safety Promotion & Injury Prevention Research Center, Injury Prevention & Trauma Network, Shahid Beheshti University of Medical Sciences, Tehran, Iran.

**Keywords:** Workplace violence, physical abuse, internship and residency, emergency service, hospital

## Abstract

**Introduction:**

Due to the stressful nature of emergency Department (ED), residents in ED are at risk of violence from patients or their associates. This study aimed to determine the prevalence of workplace violence against ED residents and the reasons for not reporting them.

**Methods:**

This cross-sectional study was conducted on ED residents of three educational hospitals, Tehran, Iran, during 2015. The national questionnaire about workplace violence was used for data gathering. In addition, prevalence of reporting the violence and the reasons for not reporting them were determined.

**Results:**

280 questionnaires were analyzed. The mean age of residents was 32.2 ± 4.6 years (58.4% female). 224 (80%) residents stated that they had not passed any educational courses on violence management. The most prevalent type of violence was verbal (90.7%) and patients’ associates (85.4%) were the most common source of aggression. The frequency of physical violence was higher in male aggressors (p = 0.001), resident age > 30 years (p = 0.044), aggressor age > 30 years (p = 0.001), and night shift (p = 0.001). The same trend was observed regarding verbal and racial-ethnic violence. There was no significant relationship between residents’ sex, resident's specialty, and presence of security and police with frequency of violence. 214 (76.4%) residents did not report the violence, and the main reasons for not reporting from their viewpoint were uselessness of reporting (37.4%) and insignificance of the violence (36.9%).

**Conclusion:**

Based on the findings of the present study more than 90% of ED residents had experienced at least one type of verbal, physical, or racial-ethnic violence during their shifts. It is necessary for residents in EDs to be trained about violence control and also report and follow these issues through legal channels.

## Introduction:

Workplace violence, which involves physical, verbal, cultural, racial-ethnic and sexual violence, is a worrying issue for every person in every workplace and its trend is increasing (1-3). World health organization (WHO), in its first world report on violence and health, described violence as: use of physical or mental power for threatening or acting against oneself, another, a group or community, causing injury, death, mental injury, retardation in growth or deprivation, or increasing the likelihood of these events. Also, WHO estimates that nearly 1.6 million people annually die because of violence worldwide (4-6). 

Recently, workplace violence in health system has become an important issue in policymaking and it is one of the most important and complex issues in the health system. The hospital staff, including nurses and physicians, are at greater risk of workplace violence (7). The probability of workplace violence for health workers and particularly nurses is even higher than prison guards or police officers (8, 9). 

The most important subsequences of violence against health workers are increase in awful incidents, the cost of retention and employment, absenteeism, tardiness and job abandonment, avoiding patients, patients’ complaints, job burnout, and mental exhaustion and also decrease in the efficiency and job performance of healthcare staff (10). 

Insults and violence at the time of providing health care for the patient not only jeopardize the physical, emotional and psychological status of the healthcare staff, but also distort the therapeutic relationship between them and patients, which ultimately results in these adverse effects having negative influence on patients’ health (11). 

In the emergency department (ED) its stressful nature and direct contact with patients and their associates increase the likelihood of violence against health workers. Disease stress, patient pain, and high latency for doctor's arrival or receipt of medication and pain relief are some factors that can exacerbate violence against ED personnel (12). 

Despite the overcrowding of ED and the unresolved issues of emergency patients, which contribute to the rise of violence in ED, the importance of this issue has not yet been widely acknowledged in Iran. It is necessary to determine the nature and causes of this violence in order to be able to plan and make policy in this regard to provide safety for healthcare staff and improve health services for patients. Therefore, this study attempts to determine the prevalence of violence from patients and their relatives to ED residents and the reasons for not reporting.

## Methods:

***Study design and setting***

This is a multi-centric, cross-sectional study that was conducted on ED residents of three educational hospitals affiliated to Shahid Beheshti University of Medical Sciences (Imam Hossein, Shohadaye Tajrish, Loqman Hakim) in Tehran, Iran. Data collection was carried out from December 2014 to November 2015. The study followed the principles of the Declaration of Helsinki and was approved by the Medical Ethics Review Board of Shahid Beheshti University of Medical Sciences. All information about the residents was kept completely confidential, and all information is released as a group without participants’ name. Study participants did not incur any costs and the study protocol did not cause any harm to participants. Written informed consent was obtained from volunteers and details and purpose of the study were disclosed.

***Participants***

Residents working in ED of the mentioned hospitals, who volunteered to participate in the study and had contact with patients were included. Residents who were reluctant to participate in the study and those with incomplete questionnaires were excluded.

***Data gathering***

The sampling method was simple (consecutive) and residents in the ED were included in a voluntary manner.

All the residents, either those who experienced violence or those who didn’t, were questioned using the national questionnaire about workplace violence in Persian language.

The questionnaire consisted of demographic data of ED residents (age, sex, marital status, average number of shifts per week, type of shifts), questions about occurrence of the verbal, physical, or racial-ethnic violence which caused by patients and their associates, and finally questions about the reactions of residents to violence and the rate of reporting violence cases and the reasons for not reporting the cases. For eliminating some cultural limitations of participants, questions related to sexual violence were deleted from the questionnaire.

In a previous study, the validation of the national questionnaire about workplace violence has been determined and confirmed through its content validity and reliability (13). The correlation coefficient of the questionnaire was 78%.

After obtaining Ethical approval, the researcher (a senior emergency medicine resident) went to the EDs for visiting residents on morning, evening or night shifts. The researcher described the study purpose, directions for participation, and information about informed consent for residents. Then, distributed the printed anonymous questionnaires among the residents. Residents returned completed questionnaires in provided envelopes, which were delivered to the researcher. It should be noted that, distribution and filling out the printed anonymous questionnaires were never done at the same time. 

## Definitions:

**Physical violence:** use of physical strength against a person or group, including beating, kicking, slapping, using cutting devices, stabbing, shooting, firing, throwing an object, spitting, pushing, scratching, beating, pulling hair, pulling, pushing, hitting, grabbing, squeezing, twisting, punching, pinching or other similar things.

**Verbal violence:** behaviors such as intimidating, insulting, humiliating and patronizing actions, cursing, screaming, ridicule, cussing, bullying, yelling at or berating a person in front of another, slurring, and bullying that are repeatedly conceived. Also, raising of fists and attempts at physical violence were defined as verbal violence.

**Racial-ethnic violence:** Any harassment, humiliation, mockery, etc. due to ethnicity, race, language, religion and place of birth that affect human dignity.

***Statistical analysis***

Data analysis was done using SPSS version 17.0 (SPSS, Inc., Chicago, IL, USA). Data are presented as frequency and percentage for qualitative variables, and mean ± standard deviation for quantitative variables with a normal distribution. Since all quantitative parameters had normal distributions according to Kolmogorov-Smirnov test, chi-2 test was used for comparison of continuous parameters. P values less than 0.05 were considered statistically significant. 

## Results:

***Baseline characteristics of participants***

344 questionnaires were distributed among ED residents, after initial examinations and removing incomplete ones, 280 questionnaires were used for analysis. The mean age of these residents was 32.2 ± 4.6 (27- 47) years (58.4% female). Table 1 shows the baseline characteristics of studied residents. It's worth noting that in 171 questionnaires, the specialty of resident was not mentioned. 224 (80%) residents stated that they had not passed any educational courses on violence management.

***Violence against participants***

The most prevalent type of violence was verbal (90.7%) and patients’ associates (85.4%) were the most common source of aggression (table 1). The frequency of physical violence was significantly higher in male aggressors (p = 0.001), resident age > 30 years (p = 0.044), aggressor age > 30 years (p = 0.001), and night shift (p = 0.001). The same trend was observed regarding verbal and racial-ethnic violence (table 2). There was no significant relationship between residents’ sex, resident's specialty, and presence of security and police with frequency of violence.

***Predisposing factors*** ***of Violence ***

Lack of information about duties of residents among people (118; 44.7%), lack of security facilities (70; 26.5%), lack of training courses regarding violence prevention (44; 16.7%), taking psychedelic drugs or alcohol by patients (31; 11.7%), and Lack of information regarding legal issues (23; 8.7%) were the most important predisposing factors of violence on the viewpoint of ED residents, respectively. 

***Preventive factors of Violence***

ED residents reported the presence of police (82; 29.8%), performing safety measures (65; 23.6%), the presence of security personnel (41; 14.9%), training the staff about violence management (25; 9.1%), restrictive measures such as penalties for aggressors (27; 9.8%), existence of instructions on dealing with violence (18; 6.5%), violence reporting system (16; 5.8%), and separating criminal patients from other patients (1; 0.4%) as the most important preventive factors of violence in ED, respectively. 

225 (80.3%) of residents declared that participating in training courses on violence control and the existence of a management system for reporting and controlling violence are so important and necessary. 

***Residents' reaction to violence***

In response to all kind of violence, the most common reactions among residents were self-defense (78; 28.9 %), invitation of attacker to relax (66; 24.4%), not taking any action (62; 23.0%), pretending nothing had happened (32; 11.9%), asking for help (15; 5.6%), sharing with colleagues (15; 5.6%), and sharing with friends and associates (2; 0.7%), respectively.

***Reporting the violence***

76.4% of residents did not report the violence, and the main reasons for not reporting from their viewpoint were uselessness of reporting (37.4%), insignificance of the violence (36.9%), and not knowing the source or system for reporting violence (15.4%), respectively (table 3).

## Discussion:

Based on the findings of the present study, more than 90% of ED residents had experienced at least one type of verbal, physical, or racial-ethnic violence during their shifts. Male aggressors, resident and aggressor age > 30 years, and night shift significantly correlated with higher frequency of violence. Uselessness of reporting and insignificance of the violence were the most important causes of not reporting.

The findings demonstrated that residents in ED were more likely to experience verbal violence (90.7%), because when patients or their associates are upset and in a stressful condition, they first show their anger as a verbal violence (insulting, ridicule, etc.), then they turn to threatening and finally show physical violence, which was 68.6% in current study. 

In Ayranci study, in Turkish EDs, most participants had experienced verbal violence and subsequent physical threats and attacks (14). In a study in Egypt, Samir et al. reported that the most common violence against medical staff in gynecology department was physical violence with 78.1% (15). In the study by Lavorie et al., EDs of 127 educational hospitals with at least 40,000 visits per year in the state of Kentucky were surveyed, and 32% of healthcare staff encountered at least one verbal violence per day, and 25% faced at least one physical violence per day (16). Senuzum et al., reported 98.5% and 19.7% for verbal and physical violence, respectively (17). These studies are consistent with the current study and show a high prevalence of violence.

In the present study, more than half of the violence had occurred on the part of patient's associates (85.4%). This seems to be related to the unnecessary presence of the patient's associates and their roaming in EDs. Also, the lack of a waiting room in EDs creates more tension between health staff and patients’ associates. Along with the findings of current study, Salemi et al., (18) and Rafati Rahimzadeh et al. (19) also found that most violence came from the patients' associates. Ayranci's study also found that the patient's associates were the source of most of the violence in ED (14). 

Contrary to the findings of the present study which demonstrated that more physical and verbal violence happened to residents with age more than 30 years, the results of Cheraghi et al. stated that lower-age medical personnel were more at risk of experiencing verbal violence (20). In the present study, there was no significant relationship between resident's sex and specialty with frequency of violence. Yet, some previous studies have shown that in all parts of the hospital, female health staff were more often victims of physical violence (21, 22). Also, male sex and age over 30 years in aggressors had a significant correlation with violence. Consentient with these findings, previous studies showed that most violence was committed by men (23, 24). Additionally, we found that night shift in EDs is the most common time of violence occurrence, which confirms the findings of previous studies (1, 23). 

From the viewpoint of the ED residents in the present study, the main predisposing factors for violence in ED was lack of information about residents’ duties among the people and lack of security facilities. Rahmani et al. showed that 67.4% of EMS personnel mentioned lack of information about their duties among people as a main cause of violence in their workplace (25). Meanwhile, in another study, ED nurses reported a lack of control over traffic of patients’ associates, lack of control on the number of patients’ associates and a lack of security staff as the most important factors that exacerbated workplace violence (26). A study in Morocco showed that the main causes of violence against medical personnel includes delayed counseling and treatment in 52% of cases, being drunk in 17% of cases and mental illness in 5% of cases of violence (10). 

In the present study, police forces, safety measures in ED and the presence of security were mentioned as the most important factors preventing violence in EDs. Therefore, this indicates the need for supportive systems to protect victims of workplace violence.

Also, the most common reaction of ED residents was self-defense, inviting aggressor to relax and taking no action. In another study on EMS personnel, the respondents' most frequent reaction to violence was inviting aggressors to relax (25). It should be noted that such collisions between emergency staff with patients and their associates eliminate mutual trust, and may lead to very unfortunate consequences. 

76.4% of residents did not report the violence and uselessness of reporting from their viewpoint, low importance of violence and not knowing a source for reporting violence were the main reasons for not reporting violence. Similar to current findings, other studies also indicated that most violence are not reported at all and cases were only reported if there was a physical impairment. Also, some believe that facing the violence is part of their jobs and do not report them (3, 17, 18). In other studies, the greatest reasons of not reporting violence was the ineffectiveness of this report and neglecting the issue by the managers (20, 27).

**Table 1:** Baseline characteristics of the studied residents (n = 280)

Variables	Number (%)
Sex	
**Male**	116 (41.6)
**Female **	163 (58.4)
Age (year)	
**≤30**	116 (41.4)
**>30**	164 (58.6)
Marital status	
**Single**	111 (45.1)
**Married **	135 (54.9)
Postgraduate year level	
**1**	82 (38.3)
**2**	76 (35.5)
**3**	56 (26.2)
Specialty *	
**Emergency Medicine **	56 (51.4)
**Surgery**	15 (14.2)
**Orthopedics**	11 (10.4)
**Pediatrics**	2 (1.9)
**Internal**	15 (14.2)
**Neurology**	3 (2.8)
**Neurosurgery**	4 (3.8)
Violence	
**Verbal**	254 (90.7)
**Physical**	162 (68.6)
**Racial-ethnic **	25 (8.9)
Aggressor	
**Patients **	164 (58.5)
**Associates **	239 (85.4)
**Co-workers**	32 (11.4)
**Others **	31 (11.1)

* Among those who reported their specialty, some ticked more than one item.

**Table 2:** Correlation of physical, verbal and racial-ethnic violence with some variables

**Variables**	**Physical**	**P**	**Verbal**	**P**	**Racial-ethnic**	**p**
**Aggressor sex**						
Male	141 (78.8 )	0.001	185 (77.4)	0.001	20 (83.3)	0.001
Female	38 (21.2)		54 (22.6)		4 (16.7)	
**Resident Age**						
≤30	72 (45.3)	0.044	92 (55.5)	0.001	11 (50.0)	0.983
>30	87 (54.7)		115 (44.5)		11(50.0)	
**Aggressor Age**						
≤30	72 (37.5)	0.001	95 (37.4)	0.001	5 (20.0)	0.001
>30	120 (62.5)		159 (62.6)		20 (80.0)	
**Shift**						
Morning	42 (19.6)	0.001	51 (17.8)	0.001	8 (33.3)	0.015
Evening	47 (21.9)		67 (23.3)		4 (16.7)	
Night	125 (58.5)		169 (58.9)		12 (50.0)	

**Table 3:** Reasons for not reporting the violence from the viewpoint of emergency department residents

**Reasons**	**Number (%)**
Thought reporting was useless	80 (37.4)
It was not important	79 (36.9)
Did not know where to report	33 (15.4)
Felt embarrassed	11 (5.1)
Blamed him/herself	11 (5.1)
Feared negative consequences	0 (0)

In the present study, most residents (80%) had not passed any training courses on violence control, and most of them asked for participation in training courses on violence control and expressed the need for a management system to report and control violence. In another study, only 22% of residents received formal or informal education on violence, and 81% of residents did not know how to report violence (23). Also, Gates et al., found that 64% of EMS staff had not passed any training courses to prevent violence during the previous 12 months, and they asked for increased security and reduced violence, and stated that this would promote job satisfaction and health service efficacy (3). 

Supervisors, managers and coworkers should not consider violence as part of residents’ job in EDs, and ignore reporting of violence. A continuous training program for residents to use different and effective methods for coping with violence is recommended. Also, accurate reporting of violence should be considered as a preventive measurement for violence, and managers must encourage staff to do so. It may be necessary to equip EDs with a system for reporting violence to quickly identify the residents at risk and those who were hurt to take measures such as counseling and prevent more harm to residents and increase the quality of health care for patients.

Violence in EDs could interfere with residents’ concentration during practice, increase the amount of medical errors, and result in losing a shift, frequent absences, disregard to the patient, loss of job satisfaction, worrying about work, refusal to attending in stressful conditions and even leaving the job, which impose a high cost on health care systems (28).

***Limitations***

This study has some limitations that restrict its application and generalization. First, these findings are based on self-reporting of residents. Second, because of psychological effects, some residents may refuse to remember or report workplace violence, particularly racial-ethnic ones. Third, residents were asked to report the occurrence of violence in the last 12 months, which could lead to bias in remembering. Sexual violence was not investigated in this study, because according to the current culture of Iranian society, most people do not talk about sexual issues and might have led to unrealistic data. 

## Conclusion:

Based on the findings of present study, more than 90% of ED residency residents had experienced at least one type of verbal, physical, or racial-ethnic violence during their shifts. Male aggressors, resident and aggressor age > 30 years, and night shift significantly correlated with higher frequency of violence. Uselessness of reporting and insignificance of the violence were important causes for not reporting. It is necessary for residents in EDs to be trained about violence control and also report and follow these issues through legal channels.
